# How biologically relevant are interaction-based modules in protein networks?

**DOI:** 10.1186/gb-2004-5-11-r93

**Published:** 2004-11-01

**Authors:** Juan F Poyatos, Laurence D Hurst

**Affiliations:** 1 Evolutionary Systems Biology Initiative, Structural and Computational Biology Program, Spanish National Cancer Center (CNIO), Melchor Fernández Almagro 3, 28029 Madrid, Spain; 2 Department of Biology and Biochemistry, University of Bath, Bath BA2 7AY, UK

## Abstract

By applying a graph-based algorithm to yeast protein-interaction networks we have extracted modular structures and show that they can be validated using information from the phylogenetic conservation of the network components. We show that the module cores, the parts with the highest intramodular connectivity, are biologically relevant components of the networks. These constituents correlate only weakly with other levels of organization. We also discuss how such structures could be used for finding targets for antimicrobial drugs.

## Abstract 2

The authors present a method to identify modules within protein-interaction networks. Phylogenetic profiles are used to determine the biological relevance of the modules.

## Background

There is a strong belief underpinning systems biology that between the individual molecules and an organism's phenotype there exist intermediary levels of organization [1]. The lowest level, and one that can be objectively defined, is that of the motif, for example a feedforward loop [2-5]. At the next level there exist putative modules within networks [6-16]. However, unlike motifs, modules are not objectively defined and are hence rather fuzzy. Moreover, even if a stringent definition or sophisticated algorithms could be envisaged, the data used to identify such modules are typically very noisy, for example, protein-protein interaction data. The central problem [17] with the notion of modules, therefore, is not identifying putative candidates but verifying which of them really reflect an important level of biological organization, rather than artifacts of the data or module-defining protocol. In addition, it would be of interest to determine the minimal information needed to identify such candidates, so that this level of organization can be readily probed, even in relatively poorly characterized systems.

Given that we could define such modules for a particular data source, for example, protein-protein interactions, there exists the further problem of understanding how modules relate to other forms of organization. Do for example, the proteins in a given module within a protein-protein interaction network show evidence of being coexpressed? Are they regulated by the same transcription factors and do they have the same level of dispensability?

Whether we can define modules in a stringent biologically relevant fashion is not just important for our understanding of the organization of biological systems. Many authors have conjectured that if modules are real they may also be more likely to contain proteins that are essential to viability. Hence, a network approach could be imagined to hone down potential drug targets such as, for instance, candidate targets for antimicrobials.

Here we ask whether phylogenetic information could be used to verify putative interaction-based modules. The assumption we make is that if a set of proteins belongs to the same module and that module has some biological relevance, then such a set should be generally conserved to act as an integrated functional unit [18,19]. Hence we should expect a genome to contain roughly all the set components or none. The extent to which we find the module components present or absent together we define as the 'phylogenetic correlation' of the module. We show that this correlation can be used to verify putative modules in a network context and that the modules identified in this way have important biological properties.

## Results and discussion

### Extracting modules in protein networks

Several network-clustering algorithms have been developed recently that make use of the local and global properties of networks [9-11]. To this end, it is helpful to represent networks as graphs, with proteins playing the role of nodes and protein-protein interactions playing the role of edges between nodes. In such graphs, the presence of modular topology could be manifested in the fact that the shortest distance, *L*, between any given node and the rest of the nodes in the graph would exhibit a similar pattern for those nodes belonging to the same module. Alternatively, modularity could also imply that proteins within a module would interact more frequently with each other than with proteins of different modules, a property characterized by high values of a generalized clustering coefficient, *C *(see Materials and methods).

**Figure 1:**
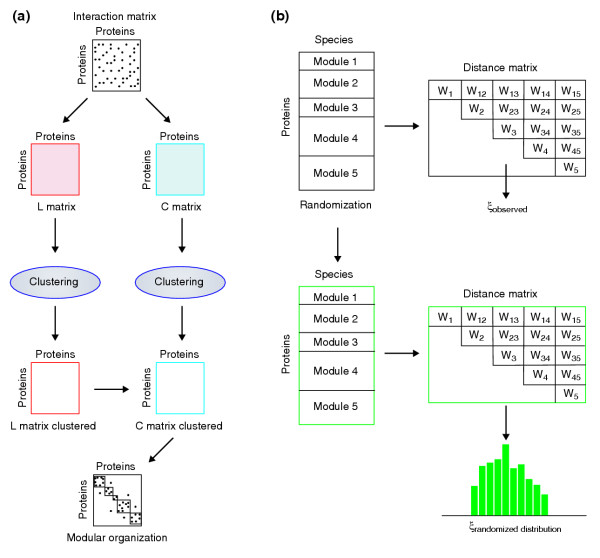
Overlap algorithm and multi-response randomization test method. **(a) **Overlap algorithm. *C*-based and *L*-based matrices are obtained from the interaction matrix. These matrices are then the input data of a standard hierarchical agglomerative average-linkage clustering algorithm [20] which extracts modules according to a given number of branches present in the clustering tree (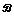) (see text). Finally, in the *C*-based modular structure, we kept in each module only those components which also appeared in the corresponding *L*-based module with which the selected *C*-module had the greatest overlap. The organization thus obtained is the putative modular organization of the network under consideration. **(b) **Multi-response permutation procedure. We validate the previous modular organization with the use of the phylogenetic conservation of module protein constituents across species. We calculate a matrix of mean pairwise similarities (or distances) among those phylogenetic profiles [18] of proteins belonging to the same module, *W_i_*, or every two pairs of modules, *W_ij_*, and computed a representative statistic *ξ*_*observed*_. *P*-values are obtained by randomly permuting the data and recomputing the statistic. This step is repeated a large number of times, 10,000 in our case. The resulting values form a randomized distribution. The observed value from the original data can then be compared with this distribution to compute the *P*-value.

We introduce here a simple algorithm that makes use of both sources of information. The basic steps of the so-called overlap algorithm are as follows (see also Materials and methods and Figure 1a).

#### Selection of the number of modules

*C*-based and *L*-based matrices were obtained from the interaction matrix. These matrices are the input data of a standard hierarchical agglomerative average-linkage clustering algorithm with a Pearson-based distance metric [20]. We obtained as an output of the clustering different sets of modules associated to each matrix by delimiting clusters according to a given number of branches present in the clustering tree (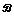) (discarding those ones containing just a single protein). In the next step we calculated an average overlap between both modular structures. A 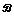-value with significantly high maximal overlap was then chosen.

#### Extraction of a particular modular structure

Having obtained *C*-based and *L*-based modules with a 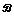-value selected as previously described, we calculated the overlap of each *C*-based module with all those obtained with the *L*-based method. An *L*-based method less efficiently discriminates modular structures in small-world networks [21], collapsing some of the modules extracted with the *C*-based technique into a unique module. The *C*-based method is more robust but is weak at discriminating modules when organization levels are high. Therefore we used the *C*-based results as a template and the *L*-based method as a filter in the extraction of modular structure. In the *C*-based modular structure we kept in each module only those components which also appeared in the corresponding *L*-based module with which the selected *C*-module had the greatest overlap. In those cases with more than one module with maximal overlap, we selected one of them at random. Although finding the optimal classification choice is a common problem of clustering analysis, this simple algorithm allows one to select a 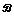-value with a high average maximal overlap and low overlap ratios between both methods, a measure of the reliability of the obtained modules (see Materials and methods and Additional data file 1 for more details).

The overlap method was applied to the yeast protein-interaction network; that is, yeast would act as an imaginary 'poorly' characterized system where we can, however, check the relevance of our findings. This was derived from two public databases (see Materials and methods) and would be, more generally, the result of high-throughput experiments. In any case, these data are probably incomplete and no doubt contain false interactions [22]. Should the analysis be done on the whole network? Certainly this could be done - and many similar analyses have been done. However, one of the novelties of the current analysis is that we perform the analysis on sub-parts. This is because we are interested in knowing whether different functional categories differ in the extent to which they might be modular [1], not least because we also want to know whether this modularity might be reflected in such things as coexpression of the genes involved. This tendency is likely to vary by functional class. For example, cell-cycle genes should in principle show a strong coexpression signal if the modules are real. In contrast, one might imagine that all cell-signaling components need to be present under all circumstances and so coexpression need not be detectable. Analyzing the network as a whole, one might come to conclude that there exists no or just a weak correspondence between modules and coexpressed genes, when in reality there might be a very strong relationship for some categories while none for others.

We therefore opted to analyze networks consisting of proteins belonging to different Munich Information Center for Protein Sequences (MIPS) protein functional categories [23]. This also has some methodological advantages. First, as methods for detecting protein-protein interactions may vary systematically according to functional grouping - for example, cytoplasmic complexes tend to be under-reported - it can be helpful to isolate each grouping alone. Second, it is probably desirable to filter out highly connected proteins to avoid big hubs and star-like clusters with low statistical significance [9]. Projecting the networks onto functional categories is a possible way of achieving such a filter. In every functional network, we found a regime of 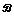-values with significantly high average maximal overlap, that is, overlap equal to or greater than 0.8, and low ratios, characterizing the reliability of the proposed modular organizations. For an analysis of the performance of the algorithm as a function of 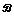-see Additional datafile 1. Note that these results extend the presence of modularity found previously in some yeast networks [9,10,24] to the functional networks introduced here. Explicit 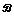-values in the regime described above were chosen such that the average module size is around 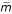 equal to 5 to 25 proteins, the so-called meso scale of biological networks [9] (Table 1).

### Modular phylogenetic profiles

To ask whether the degree of phylogenetic correlation of the modules is higher than expected, we made use of the idea of phylogenetic profiles [18]; that is, patterns of presence or absence of homologs of a given protein across different genomes. We then adapted the underlying general assumption of phylogenetic profiles, that proteins belonging to a particular functional class should display a similar pattern of homologs in a set of organisms, to a more restricted hypothesis. We considered that modules within functional networks could indeed reflect a stronger functional link among their components than with the rest of the proteins. This stronger functional link, even when all proteins in the networks are part of the same functional classification, could consequently be reflected in the correlated presence or absence of module components across different organisms - that is, their phylogenetic profiles.

To verify this initial suggestion, we examined the corresponding null hypothesis, that there is no phylogenetic correlation of the proposed structures, which is based on a completely uncorrelated distribution of phylogenetic profiles with respect to the modular organization. We made use of a class of statistical methods termed multi-response permutation procedure (MRPP). MRPPs are commonly used in ecological and environmental studies to compare an *a priori *group classification of a population in which measurements of *r *responses (*r *≥ 1) are obtained from each member of the population [25]. In contrast to well-known parametric statistical techniques such as the univariate and multivariate analysis of variance, MRPPs do not require any assumption with respect to the distribution of the response measurements. In the present case, proteins are the members of the population, modules are the group classification, and the phylogenetic profiles play the role of response measurements. A further difference from standard statistical techniques is that similarity measures, or normed distances, and not individual object measurements, are the primary units of analysis.

We compared the within-module scores to the between-module scores. For each pair of modules we calculated each between-module protein pairwise similarity and took the average of these. To examine overall between-module similarity we calculated a weighted mean correlation of all between-module similarities. We then asked about the size of the difference between the mean within-module score and the mean between-module score, that is, *ξ *=  - 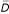 (see Materials and methods). Significance was tested by randomization; that is, we randomly permute the proteins within the modules while keeping the global modular organization fixed (Figure 1b). Not all putative network modular organizations, according to different 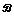
s, are shown to be biologically significant. However, we find for all networks a strong signal of phylogenetic correlation between genes in a module for some 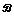-values within the regime of high reliability of the algorithm (Table 1 and Additional datafile 1).

We can extend the analysis to identify those modules showing the strongest signal. We used a method based on the analysis of each within-module similarity and the use of mean similarity dendrograms. For every module, we subtracted from the mean within-module similarity *W*_*m*_, the mean of all between-module similarity 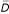, a sort of representative of all pairs of between-module similarities: that is, *ξ*_*m *_= *W*_*m *_- 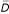. We estimated the significance of the values observed with such a modular test by performing again an approximate permutation procedure with a Holm's correction to multiple testing (Figure 1b and Materials and methods). This gives a significance measure of which module similarities reflect correlated evolution of their components in a particular functional network.

**Figure 2:**
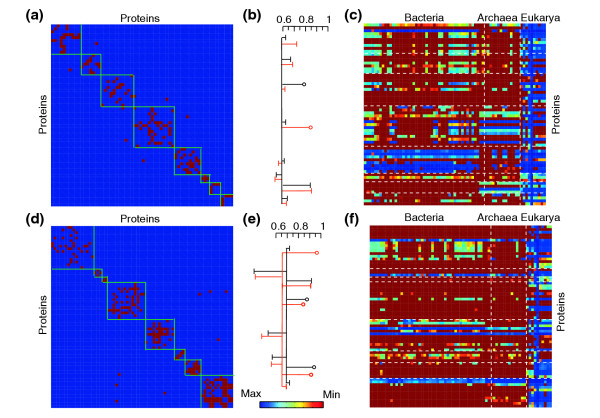
Modular organization, mean similarity dendrogram and phylogenetic profile. Modular organization, mean similarity dendrogram and phylogenetic profile of **(a-c) **cellular rescue, and **(d-f) **cellular environment functional networks. (a-d) Modular organization extracted with the network clustering algorithm. Protein interactions are plotted in brown. Modules are highlighted in white. Proteins within each module have been reorganized to show those with the greatest intra-modular connectivity - the core proteins - in the center of the module. (b,e) Mean similarity dendrograms. Branches for each corresponding module in (a) and (d) are joined at a node plotted at 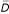. Branches terminate at the mean similarity of each module, *W_m_*, giving branch lengths of *W_m _*- 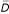 in similarity units. Dendrograms related to full modules are in black and those corresponding to the core components are in red. Those branches statistically significant (*P *< 0.05) end in a circle. (c,f) Continuous phylogenetic profiles color-coded from dark blue (maximal homology) to brown (no homology). Columns show the presence or absence of network nodes in a given organism and rows show the presence or absence of a given node in all the organism set. Species are arranged in taxonomic groups separated by white dashed vertical lines: Bacteria (left), Archaea (center), and Eukarya (right) (see Additional data file 1). The horizontal white dashed lines represent the localization of modules. A quick look at these figures provides evidence that proteins that are part of the same module exhibit a loosely correlated degree of conservation, as should be the case if modules represent some sort of discrete functional unit. This argument is quantitatively estimated by the branch length in the mean similarity dendrogram and the corresponding statistical significance.

Statistical significance does not supply any information on the magnitude of the respective similarities. To this end, we constructed a graphical representation, a mean similarity dendrogram [26], with branches for each module joined at a node plotted at 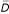. Branches terminate at *W*_*m*_, giving branch lengths of *ξ*_*m *_in similarity units (Figure 2). Those branches with considerable positive length, for example, *ξ*_*m *_equal to or greater than 0.1, indicate correlated evolution of the respective module components according to the phylogenetic profiles of the whole functional network, even though some of them could not be shown to be statistically significant because of the conservative nature of Holm's test. Thus, this combined approach provides both statistical significance and a clear quantitative picture of the compactness and isolation of the proposed modules. Figure 2 shows two examples of the application of this approach to evaluate modular network structures with the use of mean similarity dendrograms and phylogenetic profiles (we have chosen two small networks as examples to show a full picture of the modular characterization). Network phylogenetic profiles can be easily visualized as a matrix whose columns display the presence or absence of network nodes in a given organism and whose rows show the presence or absence of a given node in all the organism set. It then presents a full view of the degree of conservation of network modules for a collection of organisms. The arrangement of species in taxonomic groups is a convenient representation of the relative conservation of modules across the different lineages.

### Module cores

Previous studies suggest that any given module may have a module core and a periphery [10]. In addition, in an evolutionary context, it is not clear to what extent full modules should be present or absent in different species, considering the tinkering aspect of most evolutionary processes. Can we use the network method to discriminate a core and does the core have a stronger phylogenetic correlation? To examine this hypothesis, we selected the most connected components of each module that was part of a given network, according to their intra-modular connectivity, and applied again the overall and modular tests to these cores (see Materials and methods). We found a substantial increase in the validation of the evolutionary significance of the modules revealed, for example, by the presence of a bigger number of significant modules (Table 1, 'core' column group). Such statistically significant cores are mainly characterized by two distinct phylogenetic profiles; either their components had profiles with homologs present in all three kingdoms, or they had homologs present only in Eukarya (Table 2). This agrees with previous results and seems to support a picture of network assembly with a combination of ancient and modern modules [12,24,27].

The phylogenetic correlation suggests that this core architecture is biologically meaningful. Such extracted structures could then be used to probe this intermediate level of organization even in the case of uncharacterized biological systems. Owing to the extensive biochemical knowledge about yeast we are ready to validate such hypothesis. We have made use of the MIPs yeast complexes database [12,24] to characterize the biological relevance of the cores (see Additional data file 1 for a full list of phylogenetically distinct module cores and their biological characterization). As suggested, many, but not all, of the cores describe a significant part of relevant protein complexes, for example, anaphase-promoting complex, prenyltransferases (Ftase, GGTase I and GGTase II), some cytoplasmic translation initiation complexes such as eIF2 and eIF2B, Kel1p/Kel2p complex and Gim complexes (Table 3). Other module cores are not identified as parts of known protein complexes. This could mean either that some of the cores correspond to uncharacterized complexes or that these cores represent dynamic modules. Dynamic modules control a particular cellular activity by means of interactions of different proteins at different times or places instead of by the assembly of a macromolecular machine [1]. Thus, the combination of modular analysis and phylogenetic correlation is useful to find relevant components of biological systems.

Do we also find that the significantly phylogenetically correlated cores have other properties of biologically relevant cores, that is, show a high degree of coexpression? We examined both the extent of coexpression [28] and degree of similarity in 5' motifs [29], the latter being an indirect method of assaying possible expression parameters. As regards coexpression, most functional groups have cores with more similar coexpression than expected by chance, but the significance levels tend to be low and hence the effect, while widespread, is relatively weak. This is probably a consequence of the dynamic organization of modularity [15], a phenomenon previously observed in protein complexes [28] (Table 4 and Materials and methods). This weakness is similarly reflected in the extent of sharing of 5' motifs. This latter result is probably as expected, given a lack of certainty over the relevance of many motifs and the fact that two genes of similar expression profile can have different motifs.

Do the modules also represent units of homogeneity of dispensability? That is, if one protein in the core is lethal are all lethal, if one is dispensable are all dispensable? This can be quantified by the absolute distance of the ratio of lethal proteins in the core (0 ≤ ratio ≤1) to 1/2. We then sum these distances for the relevant cores in each network and estimate statistical significance by randomization (Figure 1b). We find some cases where there is indeed higher homogeneity than expected (Table 4). But does this also mean that the modules all contain more lethals than expected? We find that for some functional groups this is indeed very profoundly the case. However, for other functional groups this is not so (Table 4).

Assuming that the putative functional group of a protein can be assigned blind to genes, this method then has the potential to narrow down the possible drug targets in poorly described species. Perhaps as expected, cell-cycle, protein synthesis and transcription-related modules have the most significant tendency to amass lethal genes. Could we apply the knowledge of validated network structures in a therapeutical context, for instance to identify targets for antimicrobials? In principle, identifying candidate proteins as antimicrobial targets is straightforward: the protein needs to be in the microbe and not the host and to be essential to the microbe. To this end, we calculated the probability of finding lethal genes in the set of proteins without human homolog belonging to the significant cores. We compared this with the probability of finding lethal genes in those yeast proteins not found in humans which are part of the full network. While the data on which genes are essential is questionable, owing to condition-dependent lethality [30], the ratio of these two measures should give an indication of the extent to which our method improves the search strategy. Crucially, the method greatly increases the probability of finding such essential genes (Table 4). Some of these targets in yeast could be, for instance, the proteins APC4, ORC6 or POP5, which are part of complexes involved in the functional categories mentioned earlier (see Additional data file 1 for a detailed list).

## Conclusions

We have shown that by combining protein-protein data and phylogenetic information it is possible to systematically describe biologically relevant modules in protein networks which partially correlate with other types of organization. The analysis also suggests, however, that not all core modules within the functional network are equally vital for the organism's survival. This may just reflect condition-dependent lethality [30]. Indeed, the fact that fewer than half of the core metabolic modules show significant enrichment for lethal genes is possibly due to such condition-dependency. Given this result, in the development of antimicrobials it seems wiser to attack modules related to transcription, protein synthesis and the cell cycle than it is to attack metabolic pathways. This simple example hints at the relevance of knowledge about the modular organization of networks in other therapeutic settings, such as that in cancer, to home in on which modules and which parts of modules within these systems should be selected in a putative list of potential drug candidates. Overall, our results contribute to validate the relevance of the modular level of organization of biochemical networks.

## Materials and methods

### Data

We used two databases as of July 2003: MIPS [23], contributing 9,036 protein interactions; and DIP [31], contributing 15,116 interactions. Networks were assembled using a joint set of interactions after filtering common pairs. Protein information for the fully sequenced organisms selected is available at the website of the European Bioinformatics Institute [32]. A dataset on the presence of 5' regulatory motifs was downloaded from the Church Laboratory [33]. Expression data was obtained from a whole-genome mRNA expression data compiled by the Eisen laboratory [34].

### Network clustering matrices

Network clustering can be based on a global property, that is, *L*-based clustering, where *L *is referred to the shortest path length between two nodes in the network. From the interaction network, a matrix of distances is computed and transformed into an 'association' matrix by taking 1/*L*^2 ^[10]. A second approach to network clustering is based on a local property, *C*-based clustering, where *C *is a generalized local connectivity coefficient measuring common interactors of any two proteins in the interaction graph [8,9,11] given by



Here |...| denotes the size of the set, ∩ the intersection and *Adj*(*i*) the adjacency matrix, that is, the set of proteins interacting with protein *i*. Local properties tend to be more robust [11].

### Module overlap

Given two different modules, *M_i_*, *M_j_*, we considered the following overlap [13]:



with |...| denoting the size of the set and ∩ the intersection. The average overlap used to determine the number of branches present in the clustering tree (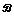) is given by:



In this case, |*C*| and |*L*| denote the number of *C*-based and *L*-based modules extracted in a given functional network.

### Network small-worldness

To characterize the small-world property of the networks, we first calculated the clustering coefficient, , and characteristic path length, *L*, for all assembled networks.  = 2*j*/*m*(*m *- 1), the ratio between the number of interactions found among the *m *proteins connected to a given one, say *j*, and the maximal potential number of such interactions, which equals *m*(*m *- 1)/2 for a undirected graph. We obtained high values of such clustering coefficient and small characteristic path length for all cases, reflecting the small-worldness of the networks. To assess the statistical significance of these values, we generated 100 randomly rewired graphs for each functional network with the algorithm described in [21]. All cases were shown to be highly significant (*P *= 0.01), that is, , and *L *≥ *L_random _*(we obtained *P *< 0.05 for *L *in the case of the energy network).

### Phylogenetic profiles

We calculated binary and continuous phylogenetic profiles [18] for different threshold values, obtaining robust results for all discussed tests in both cases. For each yeast protein of interest, BLAST searches were done against 70 proteomes of species from the Archaea (14), Bacteria (47), and Eukarya (9) (see organism list in Additional data file 1). BLAST hits with Karlin-Altschul *E*-values bigger than a given threshold, *E_th_*, were considered absent [35]. A particular value is then assigned to each homolog present, characterizing in this way every protein by means of a phylogenetic vector. For continuous profiles, homologs receive a score of -1/log*E *and the absent ones receive a score of -1/log*E_th_*. For the binary case, profiles take the value 1 or 0 when the *E*-values are below or above the threshold, respectively. Finally, note that *E*-values were corrected to account for the different database sizes. Results in the main text are for the case of binary phylogenetic profiles and a threshold value of *E_th _*= 1*e*^-6^.

### Multi-response permutation procedures

Non-parametric randomization methods, such as MRPP, have several advantages compared to more well-known parametric procedures. In particular, if the assumption of normally distributed populations is not reasonable, the datasets have multiple measurements and if multivariate comparisons are desired [25].

#### Similarity measure

Given two binary phylogenetic profiles corresponding to proteins *i*, and *j*, we considered the following matching coefficient as a simple similarity measure: *S*_*ij *_= (*x *+ *w*)/(*x *+ *y *+ *z *+ *w*), where *x *is the number of homologs present in both phylogenetic profiles, *y *is the number present in profile *i *only and *z *is the number present in profile *j *only. Finally, *w *is the number of absent homologs in both profiles.

#### Mean within and between similarities

Within similarity



Here, *c_m _*is the ratio between the number of components of module *m*, *n_m_*, and the number of components of all modules, *N_M_*, that is, *c_m _*= *n_m_*/*N_M_*, *W_m _*is the mean of similarities between proteins belonging to module *m*, and *M *is the total number of modules.

Between similarity:



Here, *c_m,s _*is the ratio between the product of the number of components of modules *m *and *s*, *n_m-_n_s_*, and the total number of components squared, *N*^2^_*M*_, that is *c_m,s _*= *n_m_n_s_*/*N*^2^_*M*_. *W_m,s _*is the mean of similarities between proteins of modules *m *and *s*, and *M *is the total number of modules. Results for all discussed tests were robust to the use of Euclidean distances with continuous profiles instead of similarities with binary profiles, as it is argued in the main text.

### Holm's test

The Holm test [36] is a method that gets round the problem of the Bonferroni procedure being too conservative, by means of the added power of sequential stepping versions of the traditional Bonferroni tests. The procedure behind the Holm test is to find all the *P*-values for a set of *k *individual tests that are being performed and then rank them from smallest to largest. While Bonferroni would compare all null hypothesis to the same value *α*, the Holm test compares the smallest to *α*/*k *and, in case of rejection of the null case, to decreasing values *α*/(*k *- 1),... until failing to reject the null.

To perform the MRPP Holm test, we computed the branch length, that is, *W_m _*- 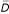 (see above) and determined the unadjusted *P*-value for each module by means of a permutation test with 10,000 randomizations. Suppose that we have *M *modules. We assemble an ordered vector of size *M *whose components are the uncorrected *P*-values in increasing order, that is, *P*_1 _is the smallest uncorrected *P*-value and *P*_*M *_is the largest. To adjust a particular vector component *P_i _*we multiply this component by *A*_i _= (*M *- *i *+ 1), thus generating a vector *P *for adjusted *P*-values. The added power of the Holm test can then be seen in a simple example. Imagine the case of three modules, that is, *M *= 3. The uncorrected *P*-values of the corresponding MRPP tests are: *P_ν _*= (0.01, 0.02, 0.03). A Bonferroni procedure for multiple testing would consider only the first test as significant according to a 0.05 significancy threshold. However, the adjusted *P*-values obtained with the Holm test would imply that all tests are significant, that is,  = *P_ν _*× (3,2,1) = (0.03, 0.04, 0.04).

### Core components

To obtain the core component of the modules, we selected for each module those components with more than two interactions, for the case of a module whose component with maximal number of interactions (MNI) is less than ten, or those components with more than four interactions for the case of a module whose component with MNI is equal to or greater than 10. Slight modifications to these rules produced similar results.

### 5' regulatory motifs, coexpression and lethality of module cores

For each of the significant module cores, *ξ*_*m*_≥ 0.1, we calculated the mean of pairwise Euclidean distances between expression vectors of proteins belonging to a given module core. In the case of the 5' motifs, the statistic measures the number of regulatory motifs common to at least more than half of the core size. Finally, for each significant core, we simply measured the number of components that are lethal. The overall statistic for all cases is the sum of each corresponding measure in each core weighted by the ratio of the core size vs network size. *P *values are obtained with 10,000 randomizations.

## Additional data files

Additional data file 1, available with the online versin of this article, includes a discussion on the network clustering algorithm, the list of species and lineages for the phylogenetic profiles, and a list of phylogenetically distinct module core components and their biological characterization.

## Supplementary Material

Additional data file 1 A discussion on the network clustering algorithm, the list of species and lineages for the phylogenetic profiles, and a list of phylogenetically distinct module core components and their biological characterization Click here for additional data file

## Figures and Tables

**Table 1:** Global and follow-up analysis of the network modular organizations

Function				Full				Core			
					
	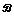	*n*	*M*	*ξ*	*P*	*P_m_*/*P_m_*^†^	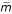	*ξ*	*P*	*P_m_*/*P_m_*^†^	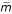
Cellular fate	34	323	14	0.012	<0.001	2/5	16.7	0.035	<0.001	3/6	6.5
Energy	25	84	5	0.066	<0.001	1/1	12.4	0.156	<0.001	1/4	4.4
Metabolism	102	420	15	0.067	<0.001	2/8	15.7	0.177	<0.001	4/9	4.7
Cellular transport	32	336	15	0.014	<0.001	2/5	18.7	0.021	< 0.001	-/2	10.8
					
Cell cycle	26	514	13	0.012	<0.001	2/3	26.6	0.05	<0.001	2/7	8.5
Protein fate	48	352	18	0.014	0.004	-/9	15.3	0.03	0.001	-/10	8.7
Transport facilitation	20	63	4	0.034	0.047	1/1	10.7	0.372	0.097	1/1	6.5
					
Cellular environment	18	87	8	0.037	0.007	2/3	8.5	0.072	0.002	3/4	5.6
Protein synthesis	16	137	7	0.038	0.002	1/1	17.3	0.194	<0.001	2/5	4.8
Cell rescue	26	88	8	0.08	<0.001	1/2	7.7	0.108	<0.001	1/3	4.2
Signaling	14	67	6	0.017	0.082	-/2	9.3	0.018	0.157	-/2	6.2
Cellular organization	36	258	15	0.032	<0.001	1/7	12.3	0.097	<0.001	3/9	5.3
Transcription	40	654	21	0.019	<0.001	2/7	25.1	0.037	<0.001	4/9	12.3

For every functional network of size *n*, we applied the network clustering algorithm with a given number of branches in the clustering tree, 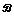. These 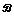-values were chosen to be among those with significantly high average maximal overlap, that is, overlap equal to or greater than 0.8, low overlap ratios, and meso-scale average module size, that is, 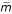 ~5-25. The outcome of this algorithm is a modular organization with *M *modules. For the follow-up analysis of both full and core components of the modules, third and fourth column groups, the following quantities are shown: *ξ*, the overall statistic, *P*, statistical significance of global test, *P_m_*^†^, number of modules whose branch length in the similarity dendrogram (see text for details) is bigger than 0.1 in similarity units and *P_m_*, number of modules whose within-similarity is statistically significant (*P *< 0.05) in the modular test. All *P*-values were obtained by means of an approximate permutation test with 10,000 randomizations and the use of binary phylogenetic profiles with a threshold of *E_th _*= 1*e*^-6 ^in the BLAST *E*-value [35].

**Table 2:** Conservation properties of module core components for those functional networks with more than one statistically significant module core

	Conservation	
	
Function	(B,A,E)	(-,-,E)
Cell fate	0(0)	6(3)
Metabolism	3(1)	6(3)
Cellular organization	3(0)	6(3)
Cellular environment	3(2)	1(1)
Protein synthesis	3(0)	2(2)
Transcription	1(1)	8(3)
Cell cycle	0(0)	7(2)

Conservation of components follows two distinct patterns: module core components are conserved in all three kingdoms: (B,A,E) Bacteria, Archaea and Eukarya, or are only present in eukaryotes, (-,-,E). The table shows the number of module cores, with branch length *ξ*_*m*_≥ 0.1, whose components have a representative phylogenetic profile of either type. Conservation profiles of statistically significant core components is shown in parenthesis. See also Table 1.

**Table 3:** List of complexes significantly represented in the phylogenetically distinct module cores

Function	Cores (r_cc _≥ 5)	Complexes
Cell fate	6 (2)	Actin-associated motor protein, 431
Energy	4 (2)	47, 346, Serine/threonine phosphoprotein phosphatase
Metabolism	9 (3)	521, GGTase II, OT
		
Cellular transport	2 (2)	Class C Vps, 239, 77, AP-3, AP-2
Cell cycle	7 (4)	Tubulins, CA, AP, 3, OR, SCF-GRR1, SCF-CDC4, RI
Protein fate	10 (5)	Vps, Class C Vps, 71, 77, FT, GGTase I, 168, 651, OT, AP, 23
Transport facilitation	1 (1)	TOM
Cell environment	4 (3)	STE5-MAPK, Kel1p/Kel2p, 521
		
Protein synthesis	5 (2)	elF3, elF2B, elF2, 340, 339, 613
Cell rescue	3 (3)	No complexes
Signaling	2 (1)	167, 308, 521
Cell organization	9 (6)	272, 5, 71, 289, casein kinase II, 181, 167, Gim
Transcription	9 (6)	154, RM, RP, Ma, Cbf, Mb, 126, NSP1, TF, 178, CPK, 634, 160, CF

Numbers correspond to those complexes found by systematic analysis as described in MIPS [23]. Abbreviations: AP, anaphase-promoting complex; CA, chromatin-assembly complex; Cbf, Cbf1/Met4/Met28; CF, core factor; CPK, cAMP-dependent protein kinase; FT, farnesyltransferase; GGTase I, geranylgeranyltransferase I; GGTase II, geranylgeranyltransferase II; Ma, Met4/Met28/Met32; Mb, Met4/Met28/Met31; OR, origin-recognition complex; OT, oligosaccharyltransferase; RI, replication initiation complex; RM, RNase MRP; RP, RNase P; TF, TFIIIC; TOM, transport across the outer membrane complex; Vps, Vps35/Vps29/Vps2. Here, *r_cc _*is the ratio between the number of complex components being part of a core and the total number of complex constituents.

**Table 4:** Statistical significance of the overall analysis of coexpression, common 5' regulatory motifs, homogeneity in dispensability and lethality for the phylogenetically distinct module cores

Function	*P*-exp	*P*-mot	*P*-hom	*P*-let	*p*-core	*p*-net
Cell fate	<0.05	-	-	<0.05	0.28	0.08
Energy	-	<.005	-	-	0	0.05
Metabolism	<0.0005	<0.05	-	<0.01	0.14	0.08
Cellular transport	-	-	< 0.01	-	None	0.28
						
Cell cycle	<0.05	-	< 0.05	0.0001	0.35	0.29
Protein fate	<0.0005	-	-	-	0.41	0.16
Transport facilitation	-	-	-	-	0.5	0.15
Cell environment	-	-	-	<0.05	0	0.06
						
Protein synthesis	<0.05	-	< 0.0005	0.0001	0.2	0.06
Cell rescue	<0.05	-	-	-	0	0.12
Signaling	-	-	-	-	0	0.12
Cell organization	<0.01	<0.05	-	-	0.08	0.12
Transcription	<0.05	<0.01	<0.01	<0.001	0.68	0.3

Statistical significance (*P*-values), of the overall analysis of coexpression (*P*-exp), common 5' regulatory motifs (*P*-mot), homogeneity in dispensability (*P*-hom) and lethality (*P*-let), for the phylogenetically distinct module cores (see text and Materials and methods for details). Not significant statistical results are denoted by -. *p*-core is the probability of finding lethal genes in the set of proteins without human homolog belonging to the significant cores. *p*-net is the probability of finding lethal genes in those proteins not found in humans which are part of each full network.
