# Supplementary material for: How biologically relevant are interaction-based modules in protein networks?
Source: Genome Biol. 2004 Nov 1;5(11):R93. doi: 10.1186/gb-2004-5-11-r93 (PMC545784; doi:10.1186/gb-2004-5-11-r93)
Supplement: Additional data file 1 — A discussion on the network clustering algorithm, the list of species and lineages for the phylogenetic profiles, and a list of phylogenetically distinct module core components and their biological characterization [file gb-2004-5-11-r93-s1.pdf]

## Supplementary Information

**1) Overlap Algorithm.** Some examples of algorithm performance for four different functional networks are shown in Fig. S1. We used the following quantities:

- Average Overlap:
  Given two different modules, $M_i$, $M_j$, we considered the following overlap [13]: $Ov_{i,j} = \frac{|M_i \cap M_j|}{\sqrt{|M_1||M_2|}}$, with $|\dots|$ denoting the size of the set and $\cap$ the intersection. The average overlap shown in Fig. S1 is given by
  $$O_C = \frac{1}{|C|} \sum_c \max\{\{Ov_{c,l}\}_{l=1\dots|L|}\}. \quad (1)$$
  In this case, $|C|$ and $|L|$ denote the number of $C$–based and $L$–based modules extracted in a given functional network.

- Overlap ratios:
  The ratio $CL$ is given by
  $$R_{CL} = \frac{1}{|C|} \sum_c |\{Ov_{c,l}\}_{l=1\dots|L|} = \max\{\{Ov_{c,l}\}_{l=1\dots|L|}\}|, \quad (2)$$
  and similarly for $R_{LC}$. All definitions as previously indicated.

High average maximal overlap indicates $\mathcal{B}$ values for which both methods extract very similar modules. Note that a decrease in the average overlap typically implies an increase in the number of different $C$–modules whose components constitute a single $L$–module, the ratio $R_{CL}$. This ratio is usually bigger than the opposite, i.e., the number of different $L$–modules whose components are part of a single $C$–module, $R_{LC}$. This is a likely consequence of the "small-worldness" of the networks under study (see Materials and Methods in main text). This property characterizes a class of networks whose *local* organization is similar to that of regular networks while its *global* organization is closer to random ones, i.e., those without any trace of modular structure [6,8,11,21]. Shortest paths among proteins in these networks would thus tend to be very similar since such property resembles the behavior of random–networks. This problem could be then only partially overcome with the introduction of the $1/L^2$ transformation in the $L$–based method. As a consequence, this method discriminates less efficiently modular structures in some conditions, collapsing in a unique module some of the modules extracted with the $C$–based technique.

We have applied this algorithm to all assembled yeast protein functional networks. In every case, we found a regime of $\mathcal{B}$ values with significantly high average maximal overlap, i.e., overlap $\geq .8$, and low overlap ratios, characterizing the reliability of the proposed modular organizations. In particular, for each of the four 4 functional groups shown in Fig. S1, we identify at least one $\mathcal{B}$ value within each group that presents a significant signal of phylogenetic correlation after Bonferroni correction.

**2) List of Species**. Organism set arranged as they are shown on Fig.1 main text (left to right, Bacteria–Archaea–Eukarya).

| ***Species*** | ***Kingdom*** | ***Lineage*** |
|---|---|---|
| Corynebacterium glutamicum | `Bacteria` | Firmicutes–Actinobacteria |
| Mycobacterium leprae | ... | Firmicutes– Actinobacteria |
| Mycobacterium tuberculosis | ... | Firmicutes–Actinobacteria |
| Streptomyces coelicolor | ... | Firmicutes–Actinobacteria |
| Anabaena | ... | –Cyanobacteria |
| Synechocystis | ... | –Cyanobacteria |
| Aquifex aeolicus | ... | – Aquificales |
| Borrelia burgdorferi | ... | –Spirochaetales |
| Treponema pallidum | ... | –Spirochaetales |
| Chlamydia muridarum | ... | –Chlamydia |
| Deinococcus radiodurans | ... | –Deinococcus |
| Thermotoga maritima | ... | –Thermotogales |
| Fusobacterium nucleatum | ... | – Fusobacteria |
| Agrobacterium tumefaciens | ... | $\alpha-$Proteobacteria–Rhizobiaceae |
| Brucella melitensis | ... | $\alpha-$Proteobacteria–Rhizobiaceae |
| Caulobacter crescentus | ... | $\alpha-$Proteobacteria–Caulobacter |
| Rhizobium meliloti | ... | $\alpha-$Proteobacteria–Caulobacter |
| Rickettsia prowazekii | ... | $\alpha-$Proteobacteria–Rickettsiales |
| Neisseria meningitidis | ... | $\beta-$Proteobacteria–Neisseria |
| Ralstonia solanacearum | ... | $\beta-$Proteobacteria–Ralstonia |
| Campylobacter jejuni | ... | $\delta/\epsilon-$Proteobacteria– |
| Helicobacter pylori | ... | $\delta/\epsilon-$Proteobacteria– |
| Buchnera aphidicola | ... | $\gamma-$Proteobacteria–Enterobacteraciae |
| Escherichia coli K12 | ... | $\gamma-$Proteobacteria–Enterobacteraciae |
| Salmonella typhi | ... | $\beta-$Proteobacteria–Salmonella |
| Salmonella typhimurium | ... | $\beta-$Proteobacteria–Salmonella |
| Yersinia pestis | ... | $\gamma-$Proteobacteria–Pasteurellaceae |
| Haemophilus influenzae | ... | $\gamma-$Proteobacteria–Pasteurellaceae |
| Pasteurella multocida | ... | $\gamma-$Proteobacteria–Pasteurellaceae |
| Pseudomonas aeruginosa | ... | $\gamma-$Proteobacteria–Pseudomonadaceae |
| Xantomonas axonopodis | ... | $\gamma-$Proteobacteria–Xanthomonas |
| Xantomonas campestris | ... | $\gamma-$Proteobacteria–Xanthomonas |
| Xylella fastidiosa | ... | $\gamma-$Proteobacteria–Xanthomonas |
| Bacillus halodurans | ... | Firmicutes–Bacillaceae |

*continued overleaf*

| ***Species*** | ***Kingdom*** | ***Lineage*** |
|---|---|---|
| Bacillus subtilis | ... | Firmicutes–Bacillaceae |
| Listeria monocytogenes | ... | Firmicutes–Listeria |
| Staphylococcus aureus | ... | Firmicutes–Staphylococcaeae |
| Lactococcus lactis | ... | Firmicutes–Streptococcaceae |
| Streptococcus pneumoniae | ... | Firmicutes–Streptococcaceae |
| Streptococcus pyogenes | ... | Firmicutes–Streptococcaceae |
| Clostridium acetobutylicum | ... | Firmicutes–Clostridiaceae |
| Clostridium perfringens | ... | Firmicutes–Clostridiaceae |
| Thermoanaerobacter tengcongensis | ... | Firmicutes–Clostridiaceae |
| Mycoplasma pulmonis | ... | Firmicutes–Mycoplasma |
| Ureaplasma parvum | ... | Firmicutes–Ureaplasma |
| Pyrobaculum aerophilum | `Archaea` | Crenarchaeota–Thermoproteales |
| Sulfolobus solfataricus | ... | Crenarchaeota–Sulfolobales |
| Sulfolobus tokodaii | ... | Crenarchaeota–Sulfolobales |
| Archaeoglobus fulgidus | ... | Euryarchaeota–Archaeoglobales |
| Halobacterium | ... | Euryarchaeota–Halobacteriales |
| Methanobacterium thermoautotrophicum | ... | Euryarchaeota–Methanobacteriales |
| Methanococcus jannaschii | ... | Euryarchaeota–Methanococcales |
| Methanopyrus kandleri | ... | Euryarchaeota–Methanopyrales |
| Methanosarcina mazei | ... | Euryarchaeota–Methanosarcinales |
| Pyrococcus abyssi | ... | Euryarchaeota–Thermococcales |
| Pyrococcus furiosus | ... | Euryarchaeota–Thermococcales |
| Pyrococcus horikoshii | ... | Euryarchaeota–Thermococcales |
| Thermoplasma acidophilum | ... | Euryarchaeota–Thermoplasmales |
| Thermoplasma volcanicum | ... | Euryarchaeota–Thermoplasmales |
| Guillardia theta | `Eukarya` | Cryptophyta–Cryptomonadaceae |
| Arabidopsis thaliana | ... | Viridiplantae–Streptophyta |
| Encephalitozoon cuniculi | ... | Fungi–Microsporidia |
| Schizosaccharomyces pombe | ... | Fungi–Ascomycota |
| Saccharomyces cerevisiae | ... | Fungi–Ascomycota |
| Caenorhabditis elegans | ... | Metazoa–Nematoda |
| Drosophila melanogaster | ... | Metazoa–Protostomia |
| Rattus norvegicus | ... | Metazoa–Deutorostomia |
| Mus musculus | ... | Metazoa–Deutorostomia |
| Homo sapiens | ... | Metazoa–Deutorostomia |

**3) List of phylogenetically distinct module cores components and their biological characterization.** Complexes shown have been selected and sorted as follows:

1) Selection: Complexes shown are those with a number of components found in the module core (CFMC) bigger than 1/4 of the core size.

2) Order: Decreasing order of the ratio, $r_{cc}$, between CFMC and the total number of *complex* constituents.

We have underlined those putative "antimicrobial" targets in the cell cycle, protein synthesis and transcription categories according to the criterion of being essential for the microbe and not found in the host. Finally, note that in the following SAC stands for Systematic Analyses Complex, according to MIPS notation [23].

*Cell fate*

1. `MYO4,SLA1,CMD1,ABP1,RVS167,RSP5,ACT1,MLC1,YSC84,MYO1, MYO3,COF1,VRP1,MYO5,SRV2,SLA2,LAS17.`

   **Actin associated motor proteins** ($r_{cc} = 0.57$)
   `MYO4,MYO1,MYO3,MYO5.`
   **SAC 387** ($r_{cc} = 0.45$)
   `MYO4,SLA1,RVS167,VRP1,LAS17.`
   **Actin associated proteins** ($r_{cc} = 0.32$)
   `SLA1,ABP1,RVS167,COF1,VRP1,SRV2,SLA2,LAS17.`
   **SAC 18** ($r_{cc} = 0.29$)
   `SLA1,YSC84,VRP1,SLA2,LAS17.`
   **SAC 17** ($r_{cc} = 0.27$)
   `MYO4,ACT1,MLC1,MYO1.`
   **SAC 294** ($r_{cc} = 0.23$)
   `MYO4,CMD1,MLC1,MYO3,COF1,MYO5.`

2. `RAS2, SPS18.` **No complex associated**

3. `CDC39,MOT2,POP2.`

   **NOT complex** ($r_{cc} = 0.4$)
   `CDC39,MOT2.`
   **CCR4 complex** ($r_{cc} = 0.23$)
   `CDC39,MOT2,POP2.`
   **SAC 263** ($r_{cc} = 0.2$)

CDC39,POP2.
**SAC 211**($r_{cc} = 0.11$)
CDC39,MOT2,POP2.

4. KAR1, BFR1, BBP1.

   **Spindle pole body** ($r_{cc} = 0.12$)
   KAR1, BBP1.

5. MUM2, KAR4. **No complex associated.**

6. SRB6,HMRA2,NUP84,MSN5,RGR1,MCM1,SIN4,GAL11.

   **SAC 431** ($r_{cc} = 1$)
   MSN5, GAL11.
   **Kornberg's mediator (SRB) complex** ($r_{cc} = 0.19$)
   SRB6,RGR1,SIN4,GAL11.
   **SAC 209** ($r_{cc} = 0.09$)
   SRB6,RGR1,SIN4,GAL11.

*Energy*

1. COX9, COX4.

   **SAC 47** ($r_{cc} = 0.5$)
   COX9, COX4.
   **Cytochrome c oxidase (complex IV)** ($r_{cc} = 0.18$)
   COX9, COX4.

2. TPS1, TPS2, TSL1,TPS3.

   **SAC 29** ($r_{cc} = 0.25$)
   TPS1, TPS2, TSL1,TPS3.
   **SAC 541** ($r_{cc} = 0.09$)
   TPS1, TSL1.

3. PDB1, LPD1.

   **Pyruvate dehydrogenase** ($r_{cc} = 0.4$)
   PDB1, LPD1.
   **SAC 42** ($r_{cc} = 0.15$)
   PDB1, LPD1.

4. GIP2,GLC7,GSY1,MDH1,GSY2,GAC1,GPH1.

   **SAC 346** ($r_{cc} = 0.83$)
   GIP2,GLC7,MDH1,GSY2,GPH1.

**Serine/threonine phosphoprotein phosphatase** ($r_{cc} = 0.5$)
GIP2,GLC7,GAC1.
**SAC 347**($r_{cc} = 0.16$)
GLC7,GSY1,MDH1,GSY2,GPH1.
**SAC 364**($r_{cc} = 0.03$)
MDH1,GPH1.

*Metabolism*

1. SCS2,SIN3. **No complex associated**
2. RTG3,CYR1,SDC25,CDC25,RAS2,INO4,RAS1.

   **SAC 521**($r_{cc} = 0.5$)
   RAS2, RAS1.
3. BET4,MRS6,BET2.

   **Geranylgeranyltransferase II (GGTase II)** ($r_{cc} = 1$)
   BET4,MRS6,BET2.
   **SAC 159** ($r_{cc} = 1$)
   BET4,BET2.
4. PRS4,PRS2,PRS3,PRS1,PRS5.

   **SAC 541** ($r_{cc} = 0.09$)
   PRS2, PRS3.
5. SRB6,SNF1,SNF4,TOR1,SSN8,SIN4,GAL11,STD1,PHO85,SSN3.

   **Kornberg's mediator (SRB) complex** ($r_{cc} = 0.14$)
   SRB6, SIN4, GAL11.
   **SAC 209** ($r_{cc} = 0.12$)
   SRB6,SSN8,SIN4,GAL11,SSN3.
6. TPS1,TPS2,TSL1,TPS3.

   **SAC 29** ($r_{cc} = 0.25$)
   TPS1, TPS2, TSL1,TPS3.
   **SAC 541** ($r_{cc} = 0.09$)
   TPS1, TSL1.
7. CDC12,BEM4,RHO1. **No complex associated**
8. OST4,LYS14,WBP1,STT3,OST5,OST1,SWP1,OST3,OST2.

**Oligosaccharyltransferase** ($r_{cc} = 0.89$)
OST4,WBP1,STT3,OST5,OST1,SWP1,OST3,OST2.
**SAC 44** ($r_{cc} = 0.44$)
WBP1,STT3,OST1,SWP1.

9. PDB1,SER3,LPD1.

   **Pyruvate dehydrogenase** ($r_{cc} = 0.4$)
   PDB1, LPD1.
   **SAC 42** ($r_{cc} = 0.15$)
   PDB1, LPD1.

*Cellular Transport*

1. VPS8, APM3, PEP3, VPS33, PEP5, AUT2, VPS16, APL5, VPS41.

   **Class C Vps protein complex**($r_{cc} = 1$)
   PEP3, VPS33, PEP5, VPS16.
   **SAC 239** ($r_{cc} = 0.67$)
   APM3, APL5.
   **SAC 77**($r_{cc} = 0.6$)
   VPS8, APM3, PEP3, VPS33, PEP5, VPS16, VPS41.
   **AP-3 complex** ($r_{cc} = 0.5$)
   APM3, APL5.
   **SAC 78** ($r_{cc} = 0.2$)
   APM3, APL5.

2. APL3, APL1, APS2, APM4.

   **AP-2 complex**($r_{cc} = 1$)
   APL3, APL1, APS2, APM4.
   **SAC 78**($r_{cc} = 0.4$)
   APL3, APL1, APS2, APM4.

*Cell Cycle*

1. TPD3,PPH21,PPH22,SIR4,CDC55,SIR3,ZDS2,ZDS1.

   **SAC 280** ($r_{cc} = 0.36$)
   TPD3,PPH21,PPH22,CDC55.
   **SAC 464** ($r_{cc} = 0.33$)
   TPD3,PPH21,PPH22.
   **SAC 584** ($r_{cc} = 0.33$)
   SIR4,SIR3.

**SAC 585** ($r_{cc} = 0.33$)
SIR4,SIR3.
**SAC 465** ($r_{cc} = 0.23$)
TPD3,PPH22,CDC55.
**SAC 170**($r_{cc} = 0.13$)
TPD3,PPH21,PPH22,CDC55,ZDS2,ZDS1.
**SAC 607** ($r_{cc} = 0.11$)
PPH21,PPH22.
**SAC 668** ($r_{cc} = 0.07$)
TPD3,PPH21,PPH22.

2. <u>RPG1</u>,PAC2,BIM1,TUB2,TUB1,PRT1.

   **Tubulins** ($r_{cc} = 0.5$)
   TUB2,TUB1.
   **elF3 complex** ($r_{cc} = 0.28$)
   RPG1,PRT1.
   **SAC 189** ($r_{cc} = 0.09$)
   RPG1,PRT1.
   **SAC 107** ($r_{cc} = 0.07$)
   RPG1,PRT1.
   **SAC 204** ($r_{cc} = 0.04$)
   RPG1,PRT1.
   **SAC 149** ($r_{cc} = 0.02$)
   RPG1,PRT1.

3. BMH2,GLC7,RED1. **No complex associated**

4. MSI1,MEC3,RLF2.

   **Chromatin assembly complex** ($r_{cc} = 0.67$)
   MSI1,RLF2.
   **SAC 430** ($r_{cc} = 0.4$)
   MSI1,RLF2.

5. CDC24,SOH1,TEM1. **No complex associated**

6. <u>CDC27</u>,APC11,<u>APC4</u>,CDC26,DOC1,CDC23,JSN1,CDC16,APC9,APC2,APC1,<u>APC5</u>.

   **Anaphase promoting complex** ($r_{cc} = 1$)
   CDC27,APC11,APC4,CDC26,DOC1,CDC23,CDC16,APC9,APC2,APC1,APC5.
   **SAC 3** ($r_{cc} = 0.86$)
   CDC27,DOC1,CDC23,CDC16,APC2,APC1.

7. ORC2,CKS1,CDC28,CDC53,CDC34,SKP1,CDC4,ORC6,CDC6,GRR1, ORC3,ORC1,ORC5,CLN2,ORC4.

   **Origin recognition complex** ($r_{cc} = 1$)
   ORC2,ORC6,ORC3,ORC1,ORC5,ORC4.
   **SCF-GRR1 complex** ($r_{cc} = 1$)
   CDC53,CDC34,SKP1,GRR1.
   **SCF-CDC4 complex** ($r_{cc} = 0.8$)
   CDC53,CDC34,SKP1,CDC4.
   **Replication initiation complex** ($r_{cc} = 0.75$)
   ORC2,ORC6,ORC3,ORC1,ORC5,ORC4.
   **Pre-replication complex** ($r_{cc} = 0.44$)
   ORC2,ORC6,CDC6,ORC3,ORC1,ORC5,ORC4.
   **Replication complex** ($r_{cc} = 0.30$)
   ORC2,ORC6,ORC3,ORC1,ORC5,ORC4.
   **SAC 10** ($r_{cc} = 0.30$)
   ORC2,ORC6,ORC3,ORC1,ORC5,ORC4.

*Protein Fate*

1. SLA1,RSP5,CHC1,YAP1802,VPS29,PAN1,PEP8,VPS35,VPS5,VPS17,LAS17.

   **Vps35/Vps29/Vps26 complex** ($r_{cc} = 1$)
   VPS29,PEP8,VPS35.
   **SAC 18** ($r_{cc} = 0.18$)
   SLA1,CHC1,LAS17.
   **Actin-associated proteins** ($r_{cc} = 0.12$)
   SLA1,PAN1,LAS17.

2. VPS8,AUT7,VPS41,TLG1,VPS45,YKT6,NYV1,PEP3,VPS33,SST2, VTI1,PEP5,TLG2,PEP12,VAM3,VPS16.

   **Class C Vps protein complex** ($r_{cc} = 1$)
   PEP3, VPS33,PEP5,VPS16.
   **SAC 71** ($r_{cc} = 0.71$)
   TLG1,VPS45,YKT6,VTI1,TLG2.
   **SAC 77** ($r_{cc} = 0.6$)
   VPS8,VPS41,PEP3,VPS33,PEP5,VPS16.
   **t-SNAREs** ($r_{cc} = 0.4$)
   TLG1,TLG2,PEP12,VAM3.

3. ERP2,ERP1,KAP123.

   **SAC 69** ($r_{cc} = 0.23$)
   ERP2,ERP1,KAP123.

**Coat complex II** ($r_{cc} = 0.18$)
ERP2,ERP1.

4. SGF29,GCN5,UBP8.

   **SAC 344** ($r_{cc} = 0.33$)
   SGF29,GCN5,UBP8.
   **SAGA complex** ($r_{cc} = 0.12$)
   SGF29,GCN5.
   **ADA complexes** ($r_{cc} = 0.12$)
   SGF29,GCN5.
   **SAC 207** ($r_{cc} = 0.10$)
   SGF29,GCN5,UBP8.
   **SAC 214** ($r_{cc} = 0.04$)
   SGF29,GCN5.

5. RAM1,CDC43,RAM2,VPS9.

   **Farnesyltransferase (FTase)** ($r_{cc} = 1$)
   RAM1,RAM2.
   **Geranylgeranyltransferase I (GGTase I)** ($r_{cc} = 1$)
   CDC43,RAM2.
   **SAC 168** ($r_{cc} = 0.67$)
   RAM1,RAM2.

6. UBP13,SAC7,SMD1,MSL1,PRP21,LEA1.

   **SAC 145** ($r_{cc} = 0.09$)
   SMD1,MSL1,PRP21,LEA1.
   **SAC 146** ($r_{cc} = 0.08$)
   SMD1,MSL1,PRP21,LEA1.
   **SAC 143** ($r_{cc} = 0.08$)
   PRP21,LEA1.
   **SAC 147** ($r_{cc} = 0.05$)
   SMD1,MSL1,PRP21,LEA1.
   **mRNA splicing** ($r_{cc} = 0.05$)
   SMD1,PRP21.

7. YPT52,YPT53,VPS21,MRS6.

   **SAC 651** ($r_{cc} = 0.57$)
   YPT52,YPT53,VPS21,MRS6.
   **SAC 780** ($r_{cc} = 0.40$)

YPT52,YPT53,VPS21,MRS6.
**SAC 345** ($r_{cc} = 0.18$)
YPT52,VPS21.
**SAC 668** ($r_{cc} = 0.04$)
YPT53,VPS21.

8. OST4,WBP1,STT3,OST5,OST1,SWP1,OST3,OST2.
   **Oligosaccharyltransferase** ($r_{cc} = 0.89$)
   OST4,WBP1,STT3,OST5,OST1,SWP1,OST3,OST2.
   **SAC 44** ($r_{cc} = 0.44$)
   WBP1,STT3,OST1,SWP1.

9. SMT3,SRP1. **No complex associated**

10. CDC27,APC11,APC4,CDC26,DOC1,CDC23,CDC16,APC9,APC2,APC1,APC5.
   **Anaphase promoting complex** ($r_{cc} = 1$)
   CDC27,APC11,APC4,CDC26,DOC1,CDC23,CDC16,APC9,APC2,APC1,APC5.
   **SAC 23** ($r_{cc} = 0.86$)
   CDC27,DOC1,CDC23,CDC16,APC2,APC1.

*Transport Facilitation*

1. TOM20,TIM17,TOM40,TOM7,TOM70,TOM22.
   **Transport across the outer membrane (TOM) complex** ($r_{cc} = 0.56$)
   TOM20,TOM40,TOM7,TOM70,TOM22.

*Cellular Environment*

1. FUS3,STE7,KSS1,PBS2.
   **STE5-MAPK complex** ($r_{cc} = 0.6$)
   FUS3,STE7,KSS1.
   **SAC 385** ($r_{cc} = 0.22$)
   STE7,KSS1.
   **SAC 384** ($r_{cc} = 0.05$)
   STE7,KSS1.

2. KEL2,KEL1.
   **Kel1p/Kel2p complex** ($r_{cc} = 1$)
   KEL2,KEL1.
   **SAC 379** ($r_{cc} = 0.15$)
   KEL2,KEL1.

3. CDC24,STE50,AKR1,GIC2,STE20,GIC1,FAR1,CDC42,STE4,BEM4. **No complex associated**

4. CYR1,SDC25,CDC25,RAS2,RAS1.

   **SAC 521**($r_{cc} = 0.5$)
   RAS2, RAS1.

*Protein Synthesis*

1. PET122,PET54,PET494.

   **Mitochondrial translation complexes** ($r_{cc} = 0.21$)
   PET122,PET54,PET494.

2. RPG1,TIF35,NOP7,RPP0,TIF34,NIP1,SUI1PRT1,TIF6,TIF5.

   **elF3 complex** ($r_{cc} = 0.86$)
   RPG1,TIF35,TIF34,NIP1,SUI1,PRT1.
   **SAC 107** ($r_{cc} = 0.3$)
   RPG1,TIF35,NOP7,TIF34,NIP1,SUI1,PRT1,TIF5.
   **SAC 767** ($r_{cc} = 0.25$)
   RPG1,TIF35,TIF34,NIP1.
   **SAC 189** ($r_{cc} = 0.19$)
   RPG1,TIF35,NIP1,PRT1
   **SAC 612** ($r_{cc} = 0.18$)
   RPG1,TIF35,TIF34,NIP1,SUI1.
   **SAC 149** ($r_{cc} = 0.06$)
   RPG1,NOP7,NIP1,PRT1,TIF6.
   **SAC 204** ($r_{cc} = 0.06$)
   RPG1,NIP1,PRT1.

3. GCD6,GCD11,GCD2,SUI2,GCN3,GCD7,CDC33.GCD1,SUI3.

   **elF2B complex** ($r_{cc} = 1$)
   GCD6,GCD2,GCN3,GCD7,GCD1.
   **elF2 complex** ($r_{cc} = 1$)
   GCD11,SUI2,SUI3.
   **SAC 340** ($r_{cc} = 0.83$)
   GCD6,GCD2,GCN3,GCD7,GCD1.
   **SAC 339** ($r_{cc} = 0.67$)
   GCD11,GCD1.
   **SAC 613** ($r_{cc} = 0.61$)

GCD6,GCD11,GCD2,SUI2,GCN3,CDC33,GCD1,SUI3.
**SAC 105** ($r_{cc} = 0.44$)
GCD6,GCD11,GCD2,SUI2,GCN3,GCD7,GCD1,SUI3.
**SAC 341** ($r_{cc} = 0.44$)
GCD6,GCD11,GCD2,GCN3,GCD7,GCD1,SUI3.
**SAC 343** ($r_{cc} = 0.25$)
GCD6,GCD11,GCD2,GCN3,GCD7,GCD1.
**SAC 333** ($r_{cc} = 0.18$)
GCD2,SUI2,GCN3,GCD7,SUI3.
**SAC 142** ($r_{cc} = 0.08$)
CDC33,SUI3. **SAC 107** ($r_{cc} = 0.07$)
SUI2,SUI.
**SAC 347** ($r_{cc} = 0.06$)
SUI2,SUI3.
**SAC 621** ($r_{cc} = 0.06$)
GCD11,CDC33.

4. MRPL16,MRPL9,MRPL19,MRPL10.

   **Mitochondrial ribosomal large subunit** ($r_{cc} = 0.09$)
   MRPL16,MRPL9,MRPL19,MRPL10.
   **SAC 108** ($r_{cc} = 0.07$)
   MRPL16,MRPL9,MRPL19,MRPL10.

5. MRPS5,MRP4.

   **Mitochondrial ribosomal small subunit** ($r_{cc} = 0.06$)
   MRPS5,MRP4.
   **SAC 104** ($r_{cc} = 0.05$)
   MRPS5,MRP4.

*Cellular Rescue*

1. AHA1,SLT2,BCK1

   **SAC 593** ($r_{cc} = 0.1$)
   AHA1,SLT2,BCK1.

2. SNZ3,YCK1,SNO1,SNZ1,SNZ2,SNO2.

   **SAC 668** ($r_{cc} = 0.04$)
   YCK1, SNO2.

3. `PRE1,RPN12,PUP2,PRE3.`

   **20S proteosome** ($r_{cc} = 0.2$)
   `PRE1,PUP2,PRE3.`
   **SAC 470** ($r_{cc} = 0.2$)
   `PRE1,PUP2,PRE3.`
   **SAC 110** ($r_{cc} = 0.08$)
   `PRE1,RPN12,PRE3.`
   **SAC 111** ($r_{cc} = 0.08$)
   `PRE1,RPN12,PRE3.`
   **SAC 736** ($r_{cc} = 0.05$)
   `PRE1,RPN12.`
   **SAC 170** ($r_{cc} = 0.04$)
   `PRE1,PUP2.`

*Signaling*

1. `GPA2,GPG1.` **No complex associated**

2. `CYR1,RAS2,SRV2,RAS1,PFY1.`

   **SAC 167** ($r_{cc} = 0.5$)
   `CYR1,SRV2.`
   **SAC 308** ($r_{cc} = 0.5$)
   `CYR1,SRV2.`
   **SAC 521** ($r_{cc} = 0.5$)
   `RAS2,RAS1.`
   **SAC 300** ($r_{cc} = 0.1$)
   `CYR1,SRV2.`
   **Actin-associated proteins** ($r_{cc} = 0.08$)
   `SRV2,PFY1.`

*Cellular Organization*

1. `SWM1,CDC23.`

   **SAC 272** ($r_{cc} = 0.67$)
   `SWM1,CDC23.`

2. `CDC24,BOI1,BEM1,GIC2,BOI2,STE20,GIC1,CDC12,CDC11,CDC42,`
   `ZDS2,CLA4,BEM4.`

   **SAC 5** ($r_{cc} = 0.75$)
   `CDC24, BEM1, BOI2.`

3. `SEC17,TLG1,VPS45,TLG2.`

   **SAC 71** ($r_{cc} = 0.57$)
   `SEC17,TLG1,VPS45,TLG2.`
   **t-SNAREs** ($r_{cc} = 0.2$)
   `TLG1,TLG2.`

4. `CKB1,CKA1,RHO3,CKA2.`

   **SAC 289** ($r_{cc} = 1$)
   `CKA1,CKA2.`
   **Casein Kinase II** ($r_{cc} = 0.75$)
   `CKB1, CKA1, CKA2.`
   **SAC 181** ($r_{cc} = 0.6$)
   `CKB1, CKA1, CKA2.`
   **SAC 631** ($r_{cc} = 0.4$)
   `CKB1, CKA1.`
   **SAC 201** ($r_{cc} = 0.23$)
   `CKB1, CKA1, CKA2.`
   **SAC 166** ($r_{cc} = 0.2$)
   `CKB1, CKA1.`
   **SAC 124** ($r_{cc} = 0.19$)
   `CKB1, CKA1, CKA2.`
   **SAC 691** ($r_{cc} = 0.17$)
   `CKB1, CKA1, CKA2.`
   **SAC 189** ($r_{cc} = 0.14$)
   `CKB1, CKA1, CKA2.`
   **SAC 608** ($r_{cc} = 0.13$)
   `CKB1, CKA1, CKA2.`
   **SAC 725** ($r_{cc} = 0.11$)
   `CKB1, CKA1, CKA2.`
   **SAC 333** ($r_{cc} = 0.11$)
   `CKB1, CKA1, CKA2.`
   **SAC 208** ($r_{cc} = 0.09$)
   `CKB1, CKA1, CKA2.`
   **SAC 288** ($r_{cc} = 0.06$)
   `CKB1, CKA1, CKA2.`
   **SAC 144** ($r_{cc} = 0.06$)
   `CKB1, CKA1, CKA2.`

5. `SLA1,ABP1,RVS167,ACT1,YSC84,SRV2,SLA2,LAS17.`

**SAC 167** ($r_{cc} = 0.5$)
ACT1,SRV2.
**SAC 233** ($r_{cc} = 0.28$)
ABP1,YSC84.
**SAC 387** ($r_{cc} = 0.27$)
SLA1,RVS167,LAS17.
**Actin-associated proteins** ($r_{cc} = 0.24$)
SLA1,ABP1,RVS167,SRV2,SLA2,LAS17.
**SAC 18** ($r_{cc} = 0.24$)
SLA1,YSC84,SLA2,LAS17.
**SAC 388** ($r_{cc} = 0.23$)
SLA1,RVS167,LAS17.

6. KAP114,NAP1,CRN1,NBP1,SVL3.

   **SAC 224** ($r_{cc} = 0.2$)
   KAP114,NAP1.
   **SAC 300** ($r_{cc} = 0.2$)
   KAP114,CRN1.
   **SAC 369** ($r_{cc} = 0.14$)
   KAP114,NAP1.
   **SAC 220** ($r_{cc} = 0.11$)
   KAP114,NAP1.

7. SKT5,CHS3,BNI4. **No complex associated**

8. GIM4,PAC10,YKE2,MEC3,GIM5,GIM3.

   **Gim complexes** ($r_{cc} = 1$)
   GIM4,PAC10,YKE2,GIM5,GIM3.
   **SAC 7** ($r_{cc} = 0.4$)
   PAC10,YKE2,GIM5,GIM3.

9. MRPS5,MRP4.

   **Mitochondrial ribosomal small subunit** ($r_{cc} = 0.06$)
   MRPS5,MRP4.
   **SAC 104** ($r_{cc} = 0.05$)
   MRPS5,MRP4.

*Transcription*

1. <u>POP5</u>,<u>POP8</u>,<u>POP7</u>,POP4,<u>SNM1</u>,<u>POP6</u>,RPP1,POP1.

   **SAC 154** ($r_{cc} = 0.87$)
   POP5,POP8,POP7,POP4,POP6,RPP1,POP1.
   **RNase MRP** ($r_{cc} = 0.78$)
   POP5,POP8,POP7,POP4,POP6,RPP1,POP1.
   **RNase P** ($r_{cc} = 0.7$)
   POP5,POP8,POP7,POP4,POP6,RPP1,POP1.
   **rRNA splicing** ($r_{cc} = 0.08$)
   SNM1,POP1.

2. MET32,<u>MET30</u>,CBF1,<u>MET4</u>,MET31.

   **Met4/Met28/Met32 complex** ($r_{cc} = 0.67$)
   MET32,MET4.
   **Cbf1/Met4/Met28 complex** ($r_{cc} = 0.67$)
   CBF1,MET4.
   **Met4/Met28/Met31 complex** ($r_{cc} = 0.67$)
   MET4,MET31.
   **SAC 413** ($r_{cc} = 0.3$)
   MET30,MET4,MET31.

3. LYS14,GTS1,SEN2,<u>SEN15</u>.

   **tRNA splicing** ($r_{cc} = 0.18$)
   SEN2,SEN15.

4. NUP42,<u>NUP145</u>,<u>NUP49</u>,CRM1,NSP1,NUP120,NUP100,GSP1,NUP116,RNA1.

   **SAC 126** ($r_{cc} = 0.67$)
   NSP1,NUP116.
   **NSP1 complex** ($r_{cc} = 0.5$)
   NUP49,NSP1.
   **SAC 441** ($r_{cc} = 0.4$)
   NUP145,NUP120.
   **SAC 545** ($r_{cc} = 0.33$)
   GSP1,RNA1.
   **NUP84 complex** ($r_{cc} = 0.33$)
   NUP145,NUP120.
   **Nucleam pore complex** ($r_{cc} = 0.29$)
   NUP42,NUP145,NUP49,NSP1,NUP120,NUP100'NUP116.
   **SAC 352** ($r_{cc} = 0.25$)
   GSP1,RNA1.

**SAC 124** ($r_{cc} = 0.12$)
NUP145,NUP120.

5. TFC3,TFC1,TFC6,TFC4.

   **TFIIIC** ($r_{cc} = 0.8$)
   TFC3,TFC1,TFC6,TFC4.
   **SAC 178** ($r_{cc} = 0.57$)
   TFC3,TFC1,TFC6,TFC4.

6. YJR119c,PPR1. **No complex associated**

7. TPK1,TPK3,TPK2.

   **cAMP-dependent protein kinase** ($r_{cc} = 0.75$)
   TPK1,TPK3,TPK2.
   **SAC 634** ($r_{cc} = 0.75$)
   TPK1,TPK3,TPK2.
   **SAC 160** ($r_{cc} = 0.67$)
   TPK3,TPK2.
   **SAC 633** ($r_{cc} = 0.37$)
   TPK1,TPK3,TPK2.
   **SAC 780** ($r_{cc} = 0.2$)
   TPK1,TPK3.
   **SAC 780** ($r_{cc} = 0.2$)
   TPK1,TPK3.
   **SAC 635** ($r_{cc} = 0.17$)
   TPK1,TPK3.
   **SAC 668** ($r_{cc} = 0.04$)
   TPK3,TPK2.

8. YCR087c-a,PXR1,SRP40. **No complex associated**

9. RRN6,RRN7,RRN11.

   **Core Factor** ($r_{cc} = 0.75$)
   RRN6,RRN7,RRN11.

**Figure S1.** Network clustering algorithm. (**A** to **D**, continuos line) Average maximal overlap (Methods) between the $C$–based and $L$–based modules as a function of the number of branches present in the clustering tree, $\mathcal{B}$. This overlap acts as a measure of the reliability of the modules obtained. An average overlap of one would indicate that both methods extracted the same module organization. (**A** to **D**, dotted and dashed line, respectively) Average number of $C$ modules with maximal overlap to each $L$ module ($R_{CL}$, dotted line) and viceversa ($R_{LC}$, dashed line) as a function of $\mathcal{B}$. Ratios of one would reflect a single match with maximal overlap for every pair of extracted $C$–based and $L$–based modules. A decrease in the average overlap typically implies an increase in these ratios, which is larger for the $R_{CL}$ case as a consequence of the small-worldness of the networks [21]. These plots are a representative behavior of that found in every functional networks studied. For each case, we calculated the significance of the extracted modular organization for three different $\mathcal{B}$ values as measured by the observed overall MRPP $\xi$ statistic (see text for further details): **(A)** Cellular fate, $P = 0.004$ ($\mathcal{B} = 30$), $P = 0.028$ ($\mathcal{B} = 50$), $P = 0.059$ ($\mathcal{B} = 70$), **(B)** Metabolism, $P < 0.001$ ($\mathcal{B} = 90$), $P < 0.001$ ($\mathcal{B} = 110$), $P < 0.001$ ($\mathcal{B} = 130$), **(C)** Protein Fate, $P = .031$ ($\mathcal{B} = 30$), $P = 0.002$ ($\mathcal{B} = 50$), $P < 0.001$ ($\mathcal{B} = 70$), **(D)** Cellular Environment, $P = 0.004$ ($\mathcal{B} = 10$), $P = 0.044$ ($\mathcal{B} = 30$), $P = 0.402$ ($\mathcal{B} = 50$). $P$–values are obtained with the application of an approximate permutation test with 10000 randomizations These results imply that not all proposed modular organizations have the same evolutionary significance according to their phylogenetic profiles.

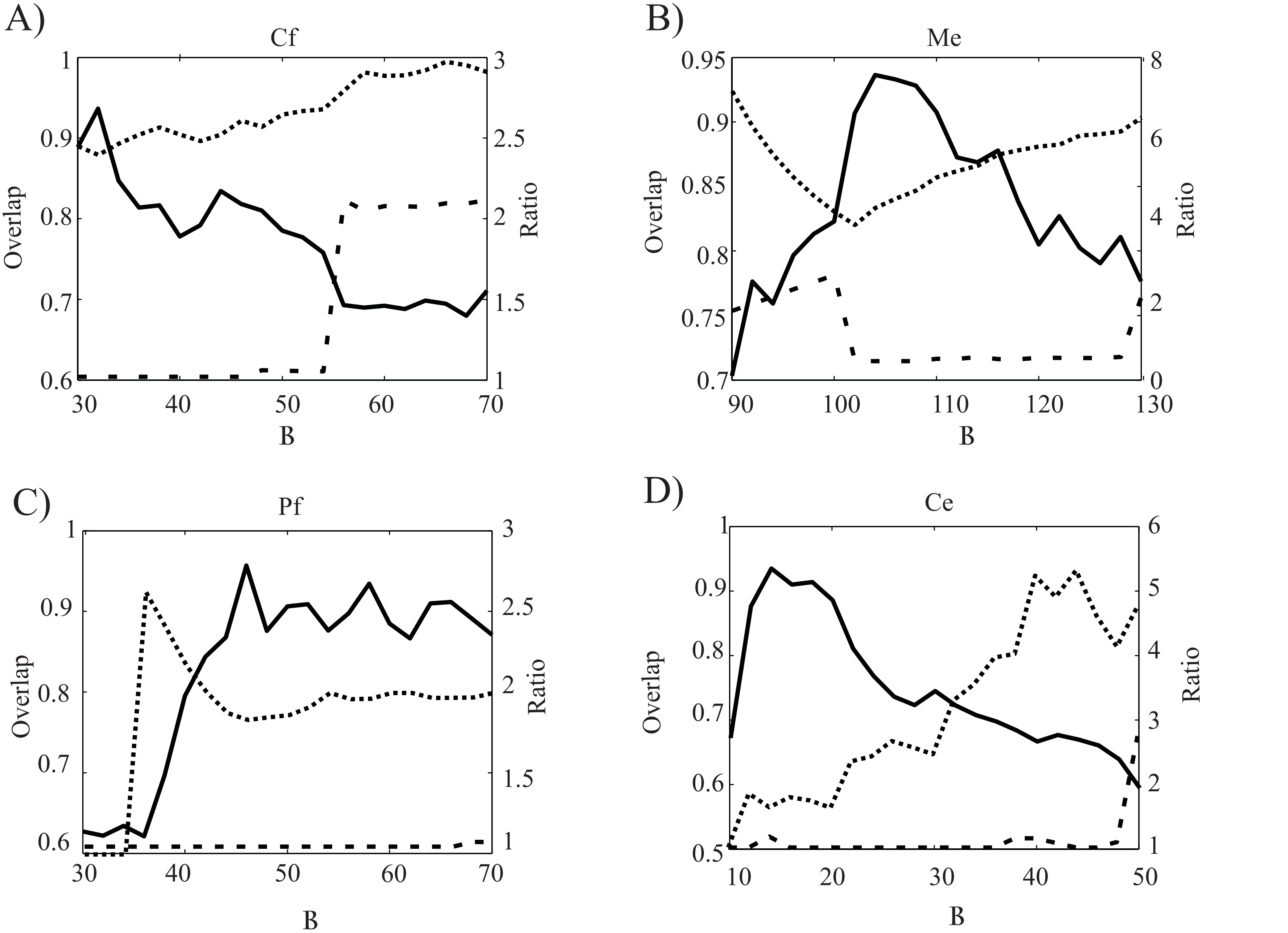

A)
Cf
Overlap
Ratio
B
B)
Me
Overlap
Ratio
B
C)
Pf
Overlap
Ratio
B
D)
Ce
Overlap
Ratio
B
